# Basilar artery plaque distribution is associated with pontine infarction and vertebrobasilar artery geometry

**DOI:** 10.3389/fneur.2023.1079905

**Published:** 2023-03-13

**Authors:** Jinmei Zheng, Bin Sun, Ruolan Lin, Yongqi Teng, Enshuang Zheng, Xihai Zhao, Yunjing Xue

**Affiliations:** ^1^Department of Radiology, Fujian Medical University Union Hospital, Fuzhou, Fujian, China; ^2^Department of Radiology, Changle District Hospital of Fuzhou, Fuzhou, Fujian, China; ^3^Department of Biomedical Engineering, Center for Biomedical Imaging Research, Tsinghua University School of Medicine, Beijing, China

**Keywords:** basilar artery atherosclerosis, plaque distribution, pontine infarction, vertebrobasilar artery, geometry

## Abstract

**Background:**

Basilar artery (BA) atherosclerosis is a common cause of posterior-circulation ischemic stroke. In this study, we investigate the relationship between BA plaque distribution and pontine infarction (PI), further, explore the influence of vertebrobasilar artery (VBA) geometries on BA plaque distribution.

**Materials and methods:**

303 patients were performed with MRI in this study, patients were divided into three groups: no cerebral infarction (NCI), anterior circulation cerebral infarction (ACCI), and posterior circulation cerebral infarction (PCCI), the VBA geometry was classified into four configurations: Walking, Tuning Fork, Lambda, and No Confluence. The AP-Mid-BA, Lateral-Mid-BA, and VA-BA angles were measured on three-dimensional time-of-flight magnetic resonance angiography. Patients underwent high-resolution magnetic resonance imaging to evaluate the BA plaque distribution (either anterior, posterior, or lateral wall). Acute and subacute cerebral infarction [including pontine infarction (PI)] were identified by T2 weighted imaging-fluid-attenuated inversion recovery and diffusion-weighted imaging.

**Results:**

The presence of BA plaque (*P* < 0.001) were associated with PCCI. Eighty-six patients all with BA plaque were further analyzed, compared with patients without pontine infarction, patients with pontine infarction were more likely to have plaque distributed at the posterior wall (*P* = 0.009) and have larger VA-BA anger (38.72° ± 26.01° vs. 26.59° ± 17.33°, *P* = 0.035). BA plaques in patients with pontine infarction were more frequently located at the posterior wall (50.00%) than at the anterior (10.00%) and lateral (37.50%) walls (*P* = 0.028). In Walking, Lambda and No Confluence geometry, BA plaques were prone to located at the lateral wall than at the anterior and posterior walls (all *P* ≤ 0.05). In the Tuning Fork group, BA plaques were evenly distributed.

**Conclusion:**

BA plaque was related to PCCI, BA plaque distribution was associated with PI, and VBA configuration strongly influences BA plaque distribution.

## Background

Posterior circulation stroke is defined by infarction occurring within the vascular territory supplied by the vertebrobasilar artery (VBA), and accounts for about 20–30% of all ischemic strokes (1, 2). Klein et al. (3) used high resolution magnetic resonance imaging (HR-MRI) to observe 41 patients with pontine infarction (PI), and found that more than 70% of patients had basilar artery (BA) plaque. BA atherosclerosis is a common cause of posterior circulation ischemic strokes, whose underlying mechanisms include artery-to-artery embolism, *in situ* thrombo-occlusion, and hemodynamic impairment (4). In addition, plaques located near the branch artery orifices may also induce pontine infarction. Previous studies have demonstrated that coronary and middle cerebral artery atherosclerosis plaques tend to be distributed at positions opposite to the orifices of the perforating artery (5, 6). The BA forms the central core of the posterior circulation and is a rich source of perforating arteries; therefore, it is meaningful to investigate the relationship between BA plaque distribution and pontine infarction.

Usually, the vertebrobasilar system is formed by the basilar and bilateral vertebral arteries. As the diameters of the left and right VAs and their anatomical course are different, a study by Yu et al. (7) classified the vertebrobasilar system into four geometric configurations: Walking, Tuning Fork, Dominant-Lambda, and Hypoplasia-Lambda. Furthermore, they (7) demonstrated that the geometric configurations of the vertebrobasilar artery strongly influence BA plaque locations. Wake-buck et al. (8) reported a relationship between vertebrobasilar geometries and differences in hemodynamic distribution. In the study by Zheng et al. (9), VBA geometry was qualitatively classified into four basic geometric configurations: Walking, Tuning Fork, Lambda, and No Confluence and found that the Walking, Lambda, and No Confluence geometry are associated with the presence of BA plaque. In this study, we want to investigate the relationship between BA plaque distribution and pontine infarction, further, explore the influence of vertebrobasilar artery geometries (Walking, Tuning Fork, Lambda, and No Confluence) on BA plaque distribution.

## Materials and methods

### Patients

This study enrolled 303 consecutive patients who presented with neurologic deficit symptoms and suspected posterior circulation ischemic stroke or transient ischemic attack to the Department of Neurology of our hospital from July 2017 to June 2018. Acute/subacute cerebral infarction (ASCI) was defined when patients presented as sudden neurologic dysfunction caused by focal brain ischemia with imaging evidence showing heperintensity on diffusion-weighted image (DWI) and corresponding hypointensity on apparent diffusion coefficient (ADC) maps, along with T2-weighted fluid attenuated inversion recovery imaging (T2WI-FLAIR) hyperintensity. While an ischemic episode with neurologic deficits but without acute/subacute infarction defines transient ischemic attack (TIA) (10). The inclusion criteria were (1) suspected ischemic stroke or transient ischemic attack in the posterior circulation; (2) imaging quality is sufficient for analysis. The details of the inclusion criteria and clinical characteristics of the participants were described in the previous study (9). The exclusion criteria were (1) non-atherosclerotic vasculopathy, such as dissection, arteritis, or Moya-Moya disease; (2) contraindications to MR imaging; (3) evidence of a cardioembolic stroke (atrial fibrillation); (4) poor image quality due to motion artifact. Seven patients with severe motion artifacts were excluded, 296 patients were included in the analysis.

To explore the association between the presence of BA plaque and posterior circulation cerebral infarction (BA-PCA region), five patients have cerebral infarction in both anterior circulation and posterior circulation (BA-PCA region), seven patients have cerebral infarction in the region supplied by posterior inferior cerebellar artery (PICA) and two patients have medullary infarction were excluded in this part, 282 patients were analyzed in the Table 1.

**Table 1:** Association between the BA plaque and cerebral infarction.

	**NCI (*n* = 203)**	**ACCA (*n* = 49)**	**PCCI (*n* = 30)**	***P***
Sex, male	120 (59.1)	31 (63.3)	20 (66.7)	0.672
Age, y	70.0 (62.0–76.0)	70.0 (57.5–79.0)	65.4 ± 11.0	0.382
Smoking	66 (32.5)	22 (44.9)	14 (46.7)	0.121
BMI ≥ 28	24 (11.8)	8 (16.3)	4 (13.3)	0.695
Hypertension	135 (66.5)	39 (79.6)	23 (76.7)	0.139
Diabetes mellitus	67 (33.0)	19 (38.8)	11 (36.7)	0.719
Hyperlipidemia	77 (37.9)	24 (49.0)	15 (50.0)	0.214
CHD	28 (13.8)	4 (8.2)	2 (6.7)	0.350
BA plaque	43 (21.2)	17 (34.7)	19 (63.3)	< 0.001^a^
Plaque slices	0 (0–0)	0 (0–5.5)	3 (0–10)	< 0.001^b^

Values are presented as mean ± standard deviation or number (%); BMI, body mass index; CHD, coronary heart disease; BA, basilar artery; NCI, no cerebral infarction; ACCI, anterior circulation cerebral infarction; PCCI, posterior circulation cerebral infarction.

^a^NCI vs. ACCI, *P* = 0.046; NCI vs. PCCI, *P* < 0.001; ACCI vs. PCCI, *P* = 0.013. the significant level was adjusted to *P*′, *P*′ = *P*/m, m = k (k−1)/2 + 1, k indicates the group number, and *P*′ < 0.013 considered to be statistically significant.

^b^NCI vs. ACCI, *P* = 0.144; NCI vs. PCCI, *P* < 0.001; ACCI vs. PCCI, *P* = 0.021.

**Figure 1:**
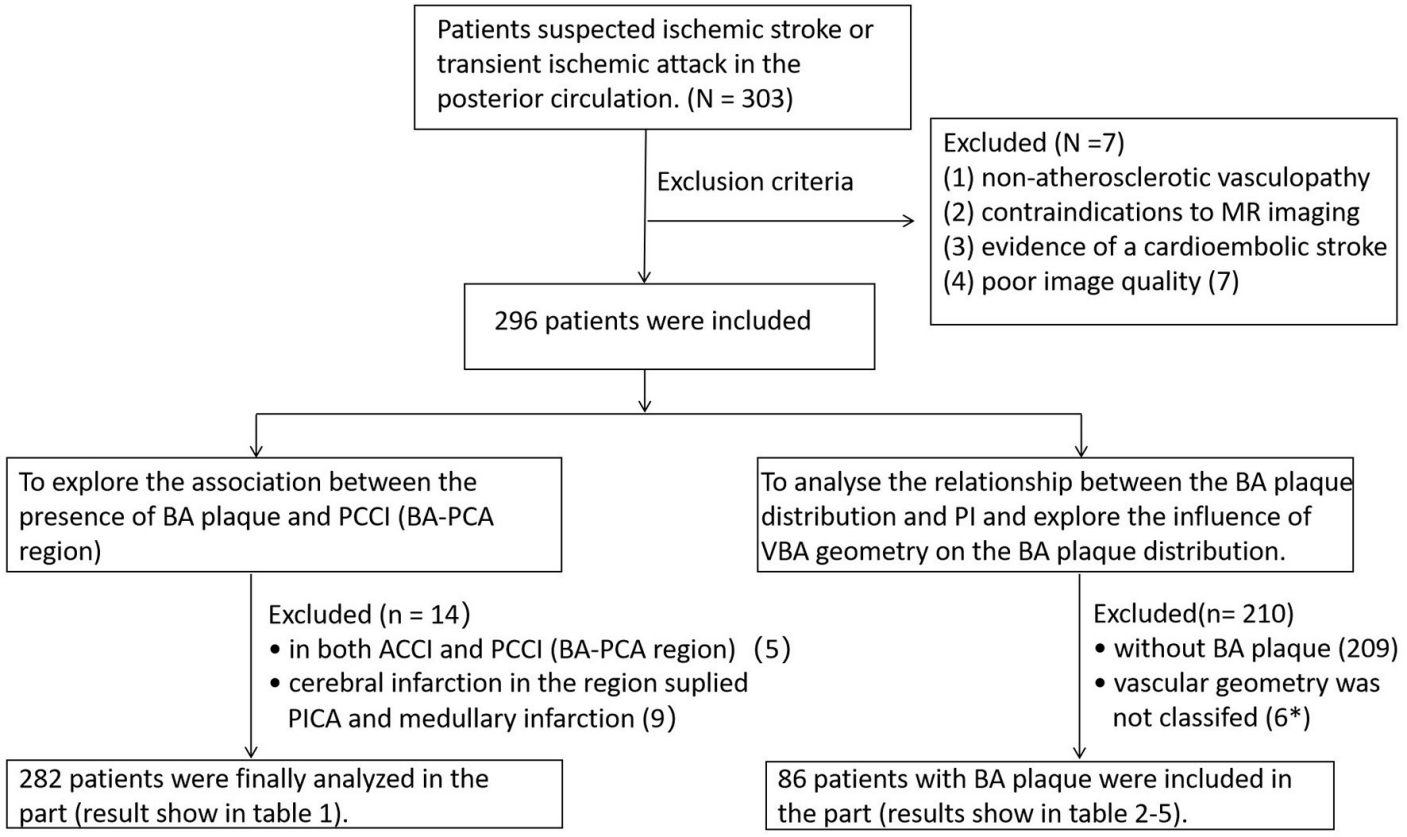
Flow diagram of patient enrollment. ^*^Six patients vascular geometry were not classified, and five patients without BA plaque. BA, brasilar artery; VBA, vertebrobasilar artery; PI, pontine infarction; ACCI, anterior circulation cerebral infarction; PCCI, posterior circulation cerebral infarction; PCA, posterior cerebral artery; PICA, posterior inferior cerebellar artery.

To analyze the relationship between the BA plaque distribution and PI, further explore the influence of VBA geometry on the BA plaque distribution, 209 patients without BA plaque and six patients in whom vascular geometry could not be classified were excluded in this part, five patients whose vascular geometry failed to classify have no BA plaque. Finally, 86 patients all with BA plaque were included in the Tables 2–**5**. Flow diagram of patient enrollment was show on Figure 1.

**Table 2:** Comparisons of clinical risk factors between patients with PI and without PI.

	**PI (+) (*N* = 13)**	**PI (–) (*N* = 73)**	***P***
Sex, male	10 (76.92)	40 (54.79)	0.222
Age, y	67 (60.5–75.5)	73 (63.5–79.0)	0.136
Smoking	6 (46.15)	23 (31.51)	0.303
BMI ≥ 28	2 (15.38)	15 (20.55)	1.000
Hypertension	11 (84.62)	58 (79.45)	1.000
Diabetes mellitus	5 (38.46)	34 (46.58)	0.588
Hyperlipidemia	7 (53.85)	31 (42.47)	0.447
CHD	2 (15.38)	9 (12.33)	0.670
AP-mid-BA angle (°)	19.40 (13.80–38.35)	27.40 ± 16.40	0.527
VA-BA angle (°)	38.72 ± 26.01	26.59 ± 17.33	0.035
Lateral-mid-BA angle (°)	23.59 ± 12.88	22.91 ± 13.66	0.868

Values are presented as numbers (%) or medians (interquartile ranges).

PI, pontine infarction; BMI, body mass index; CHD, coronary heart disease.

This study was conducted following the Declaration of Helsinki and was approved by the Ethics Committee of Union Medical College Hospital Affiliated to Fujian Medical University. All patients signed an informed consent form to participate in the research.

### Image analysis

**Figure 2:**
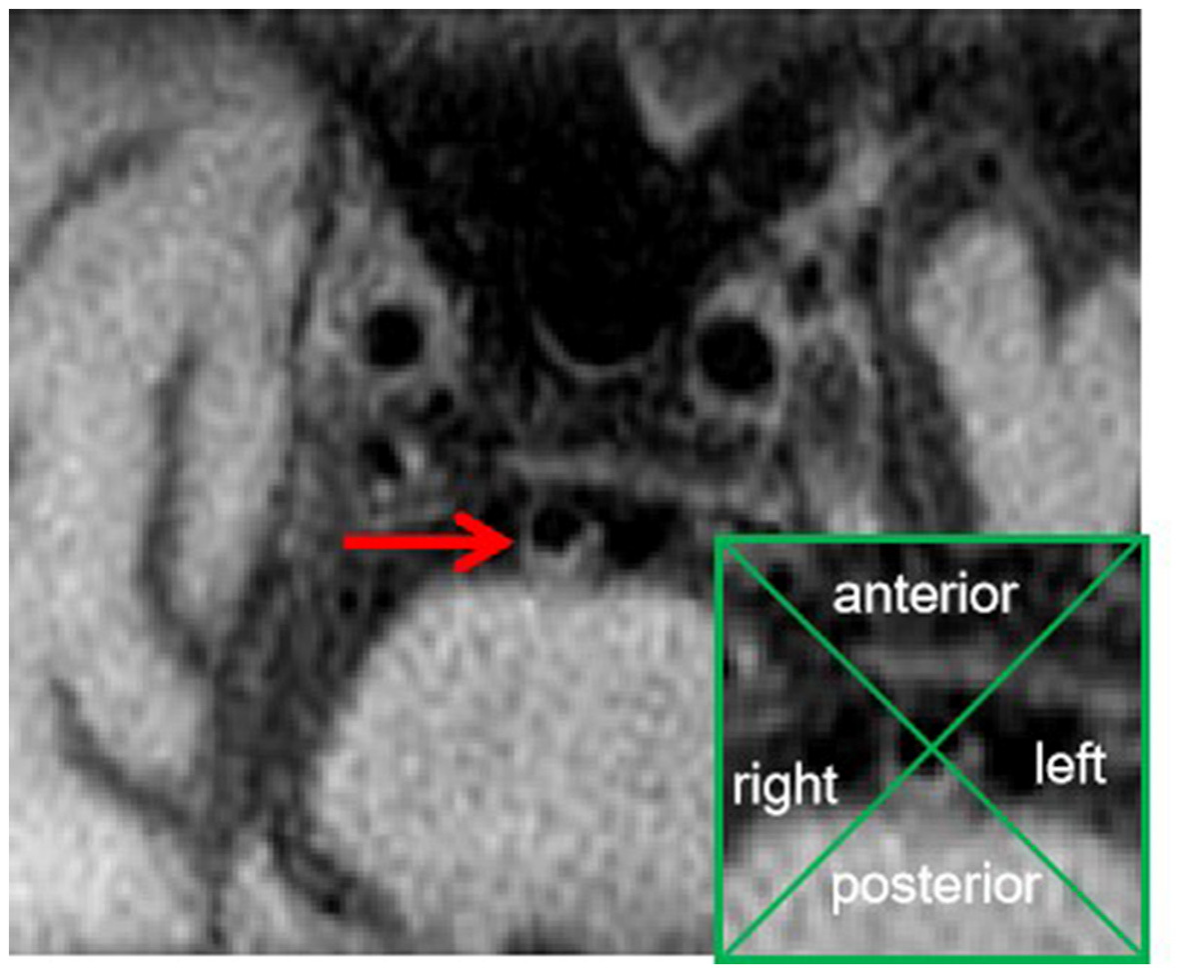
An alignment grid to demonstrate how each cross-section is divided into four quadrants.

Details of the high -resolution MRI protocol were described elsewhere (9). On the axial CUBE images, if there was eccentric wall thickening, whereas the thinnest part was estimated to be < 50% of the thickest point by visual inspection, then, a plaque was identified (6). All cross-sections with eccentric plaque were classified based on their plaque orientation being centered on the anterior, posterior, or lateral (left or right) sides of the vessel (Figure 2) (6, 11). Each cross-section was grouped into one of four quadrants. In cases where the plaque was located at more than one quadrant, the quadrant with the maximal plaque thickness was chosen (6, 11), and we calculated the percentage of plaque distribution at the anterior, posterior, and lateral sides of the basilar wall.

Acute and subacute cerebral infarction (including pontine ischemic lesions) were identified by T2WI-FLAIR and DWI. Patients were divided into three groups: no cerebral infarction (NCI), anterior circulation cerebral infarction (ACCI), and posterior circulation cerebral infarction (PCCI). ACCI is defined as atherosclerotic cerebral infarction in the region of the internal carotid artery system. PCCI mean cerebral infarction of the vertebrobasilar artery system [BA-posterior cerebral artery (PCA) region], excluding cerebral infarction in the region supplied by posterior inferior cerebellar artery (PICA) and medullary infarction in this study.

The diameter of the VA was measured using three-dimensional time-of-flight magnetic resonance angiography (TOF-MRA). We measured three consecutive points, 3 mm apart, starting from the vertebrobasilar junction (both VAs and the BA), then, the diameter of each vessel was calculated as the average of the three measurements values (7, 12). The dominant vertebral artery was defined as the vertebral artery with the widest diameter (difference in diameter ≥ 0.3 mm) (7, 12). Based on the TOF-MRA images, the vertebrobasilar artery geometry was group into four configurations: Walking, Tuning Fork, Lambda, and No Confluence. The characteristics of each geometric configuration was defined elsewhere (9). The VBA geometry was measured by two experienced readers (Z.J. and X.Y.). The differences between the two observers were solved by consensus. To assess intra-observer reproducibility, TOF-MRA images were reevaluated by one reviewer (Z.J.) 1 month later.

**Figure 3:**
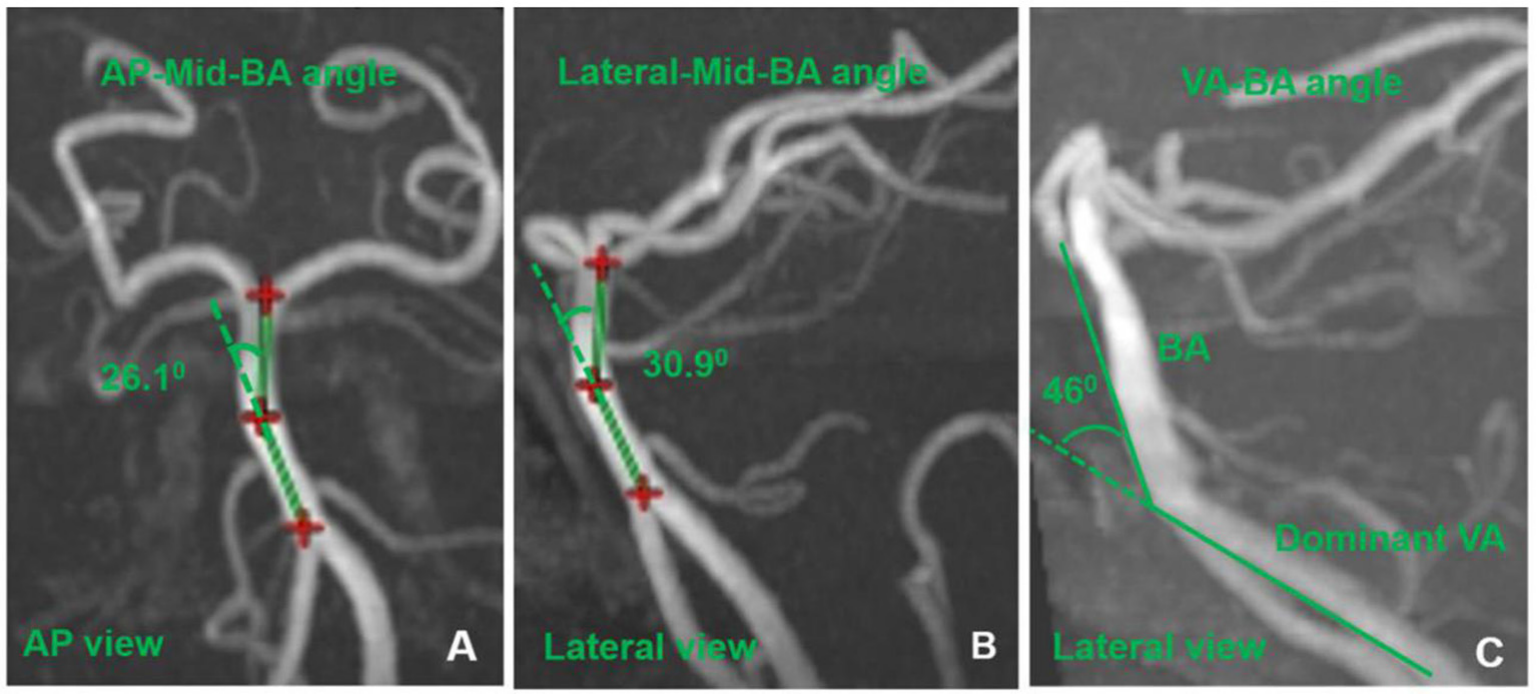
The measurement of AP-mid-BA angle **(A)**, lateral-mid-BA angle **(B)**, and VA-BA angle **(C)**. BA, basilar artery; VA, vertebral artery.

The AP-Mid-BA angle was measured using the anteroposterior (AP) view of 3D-reconstructed TOF-MRA, and the vertebral artery-basilar artery (VA-BA) angle and Lateral-Mid-BA angle were measured using the lateral view of the 3D-reconstructed TOF-MRA (Figure 3). Imaginary lines were drawn from the mid-BA to the vertebrobasilar junction and the top of the BA in the AP and the lateral views, respectively (Figures 3A, B) (13). The maximum angles between these two imaginary lines were considered to be the AP-Mid-BA angle (AP view) and the Lateral-Mid-BA angle (lateral view). Using the same method, imaginary lines were drawn from the vertebrobasilar junction to the BA and the dominant VA; the angle between the two lines was considered the VA-BA angle (Figure 3C) (13).

### Statistical analysis

Statistical analysis was performed using IBM SPSS statistics software (version 19.0, IBM Corp., Armonk, NY, USA). The Shapiro–Wilk test was used to test whether the data met the normal distribution. Intra-observer or inter-observer variability for VBA geometry measurements was performed using intraclass correlation coefficient (ICC) analysis. Quantitative data are expressed as mean ± SD, and qualitative data are expressed as percentages. The comparison of BA plaque prevalence among patients with NCI, ACCI, or PCCI was performed using chi-square test. Comparison of BA plaque slices among the three cerebral infarction groups was performed by the Kruskal-Wallis test. Comparisons of BA plaque distribution incidence in the anterior, posterior, and lateral sides of the BA wall were performed using Friedman's M test. Comparisons of plaque distribution incidence between patients with and without pontine infarction were performed using the Mann-Whitney *U*-test, and data comparisons of the four geometric configurations were performed by variance analysis or the Kruskal-Wallis test. *P* < 0.05 was considered statistically significant.

## Results

Seven patients with severe motion artifacts were excluded. In the 296 patients, 55 patients with anterior circulation cerebral infarction (among the 55 patients, five patients with PCCI, one patient with medullary infarction), 35 patients with posterior circulation cerebral infarct, seven patients with cerebral infarction in the area of PICA, one patient only had medullary infarction, 203 patients had no cerebral infarction.

Seven patients with cerebral infarction in the region of PICA, two patients with medullary infarction were excluded, other five patients with cerebral infarction both in the anterior and posterior circulation also were excluded, 282 patients were finally analyzed in the Table 1. Table 1 shows that the presence of basilar artery plaque and the slices of basilar artery plaque were associated with the cerebral infarction of vertebrobasilar artery system (BA-PCA region) (all *P* < 0.001).

In the 296 patients, 87 patients had BA plaque. One patient with BA plaque, whose vascular geometry could not be classified, was excluded. Ultimately, 86 patients with BA plaque were included in the next analysis (Tables 2–**5**). The details of clinical information of the 86 patients were described elsewhere (9).

Of the 86 patients, there were 13 patients (median age, 67 years) with pontine infarction, and 73 patients without pontine infarction (median age, 73 years). Comparisons of clinical risk factors between patients with and without pontine infarction are summarized in Table 2. Compared with patients without pontine infarction, patients with pontine infarction were more likely to have larger VA-BA angle (38.72° ± 26.01° vs. 26.59° ± 17.33°, *P* = 0.035).

The inter-observer and intra-observer reproducibility for VBA geometry measurement were ICC = 0.851 [95% confidence interval (CI) 0.667–0.938] and ICC = 0.872 (95% CI 0.706–0.947), respectively.

### Correlation between BA plaque distribution and pontine infarction

In 86 patients, plaques were identified in 717 slices. Only four patients had a one-slice plaque; most BA plaques involved multiple slices on high resolution magnetic resonance imaging (HR-MRI). The average length of BA atherosclerosis plaque was 8.00 mm (4.50–11.00 mm) in 73 patients without pontine infarction and 9.00 mm (4.00–11.50 mm) in 13 patients with pontine infarction (*P* = 0.570).

BA plaques were more frequently located at the lateral walls than at the anterior and posterior walls in all 86 patients as well as in the 73 patients without pontine infarction. However, in the pontine infarction group, plaques were more frequently located at the posterior wall (50.00%) than at the anterior (10.00%) and lateral walls (37.50%, *P* = 0.028). Compared with patients without pontine infarction, patients with pontine infarction were more likely to have plaque distributed at the posterior wall (*P* = 0.009). The results of the basilar artery plaque distribution are shown in Table 3. The correlation between BA plaque distribution and pontine infarction was show on the Supplementary Figure 1.

**Table 3:** Basilar artery plaque distribution.

	**Anterior wall**	**Posterior wall**	**Lateral wall**	P^*^
All patients (*N* = 86)	19.09% (0–42.86%)	27.92% (0–50.00%)	48.53% (33.33–66.67%)	< 0.001^a^
PI (+) (*N* = 13)	10.00% (0–38.18%)	50.00% (23.64–66.67%)	37.50% (31.67–54.17%)	0.028^b^
PI (–) (N=73)	20.00% (0–50.00%)	22.22% (0–46.06%)	50.00% (33.33–66.67%)	< 0.001^c^
*P^#^*	0.352	0.009	0.150	

Values are presented as medians (interquartile ranges).

P^*^ indicates comparisons in the anterior, posterior, and lateral walls with Friedman's M test; *P*^#^ indicates comparisons between patients with and without pontine infarction with the Mann-Whitney *U*-test; PI, pontine infarction.

^a^Anterior vs. posterior, *P* = 0.910; anterior vs. lateral, *P* < 0.001; posterior vs. lateral, *P* = 0.002.

^b^Anterior vs. posterior, *P* = 0.056; anterior vs. lateral, *P* = 0.118; posterior vs. lateral, *P* = 1.000.

^c^Anterior vs. posterior, *P* = 1.000; anterior vs. lateral, *P* < 0.001; posterior vs. lateral, *P* < 0.001.

### Correlation between BA plaque distribution and VBA geometry

The percentage of plaque distribution at the anterior (13.64%), posterior (37.50%), and lateral walls (37.50%, *P* = 0.264) was similar in the 11 patients with Tuning Fork geometry. However, in the Walking, Lambda, and No Confluence groups, plaques were more frequently located at the lateral wall than at the anterior and posterior walls (all *P* < 0.05, Table 4).

**Table 4:** Vertebrobasilar geometric configuration and BA plaque distribution.

	**Anterior wall**	**Posterior wall**	**Lateral wall**	P^*^
Walking (26)	15.34% (0–42.86%)	27.92% (0–51.79%)	47.73% (32.50–66.67%)	0.019^a^
Tuning Fork (11)	13.64% (0–50.00%)	37.50% (0–66.67%)	37.50% (0–90.91%)	0.264
Lambda (40)	25.00% (0–50.00%)	24.35% (2.94–48.44%)	48.53% (34.38–63.35%)	0.005^b^
No Confluence (9)	11.11% (0–33.18%)	30.00% (12.42–50.00%)	50.00% (36.51–80.00%)	0.048^c^
*P^#^*	0.763	0.883	0.814	

Values are presented as medians (interquartile ranges).

P^*^ indicates comparisons in anterior, posterior, and lateral sides of the BA wall with the Friedman' M test; *P*^#^ indicates comparisons in four groups with the Wallies H test; BA, basilar artery.

^a^Anterior vs. posterior, *P* = 1.000; anterior vs. lateral, *P* = 0.025; posterior vs. lateral, *P* = 0.249.

^b^Anterior vs. posterior, *P* = 1.000; anterior vs. lateral, *P* = 0.036; posterior vs. lateral, *P* = 0.013.

^c^Anterior vs. posterior, *P* = 0.716; anterior vs. lateral, *P* = 0.055; posterior vs. lateral, *P* = 0.716.

The AP-Mid-BA angle in patients with the Walking configuration (36.13° ± 18.27°), Lambda configuration (24.84° ± 13.02°), and No Confluence configuration (24.17° ± 10.54°) were higher than that in patients with Tuning Fork configuration (14.95° ± 11.66°) (*P* = 0.001); however, there were no significant differences in the VA-BA and Lateral-Mid-BA angles among the four vertebrobasilar geometries (*P* > 0.05, Table 5).

**Table 5:** Comparisons of angle in the four geometric configurations.

	**Walking (*N* = 26)**	**Tuning Fork (*N* = 11)**	**Lambda (*N* = 40)**	**No Confluence (*N* = 9)**	***P***
AP-mid-BA angle (°)	36.13 ± 18.27	14.95 ± 11.66	24.84 ± 13.02	24.17 ± 10.54	^*^0.001^a^
VA-BA angle (°)	31.05 (13.7–42.98)	17.9 (11.6–28.6)	24.7 (18.4–37.88)	24.9 (18.95–33.4)	^#^0.362
Lateral-mid-BA angle (°)	25.40 ± 14.45	22.35 ± 10.3	21.6 ± 14.63	23.26 ± 8.5	^*^0.739

Values are presented as mean ± standard deviation, or median (interquartile range).

^*^Comparisons were performed by variance analysis.

^#^Comparison was performed by the Wallies H test.

AP, anteroposterior; VA, vertebral artery; BA, basilar artery.

^a^Walking vs. Tuning Fork, *P* < 0.001; Walking vs. Lambda, *P* = 0.003; Walking vs. No Confluence, *P* = 0.036; Tuning Fork vs. Lambda, *P* = 0.048; Tuning Fork vs. No Confluence, *P* = 0.160; Lambda vs. No Confluence, *P* = 0.900.

## Discussion

In this study, it was observed that BA plaque was associated with the cerebral infarction of basilar artery to posterior cerebral artery region. Furthermore, we found that BA plaque in patients with pontine infarction was predominantly distributed in the posterior wall. Comparatively, BA plaques in patients without pontine infarction were more frequently distributed in the lateral wall (50.00%) than in the anterior (20.00%) and posterior (22.22%) walls (*P* ≤ 0.001). Vertebrobasilar geometric configurations influence plaque distribution.

We found that BA plaque was related to the posterior circulation cerebral infarction (BA-PCA region), 19 (19/30, 63.3%) patients had BA plaque among the included 30 patients with cerebral infarction of the vertebrobasilar artery system (BA-PCA region), the result was slightly lower than the previous report (3) which indicated that more than 70% patients had BA plaque in 41 patients with pontine infarction. In our study, some cases with cerebral infarction in the PCA area, plaque may located in PCA, and BA had no plaque.

In a study by Yu et al. (11), BA plaques in the dorsal (posterior) and lateral walls were found to be associated with posterior circulation ischemic stroke, where the branches of BA originate, and that BA plaques in asymptomatic patients were more likely located at the ventral (anterior) wall. In patients with pontine infarction, we found that BA plaque was predominantly distributed in the posterior wall (50.00%), this finding is in line with previous study (11). However, in our study, the percentage of BA plaque distribution in the lateral wall (50.00%) was higher than in the anterior (20.00%) and posterior (22.22%) walls in patients without pontine infarction. BA plaque in the inferior lateral wall or in the upper lateral wall all were defined as BA plaque of the lateral wall. However, as we all know, the penetrating artery of BA arises from the posterior and inferior lateral wall, thus, BA plaque in the inferior lateral wall more easily induced pontine infraction than in the upper lateral wall. In our study, BA plaque only in the upper lateral wall accounts for about 25% of BA plaque in the lateral wall, which may be explain the result that patients without pontine infarction have high incidence of BA plaque distribution in the lateral wall.

A previous study (7) observed that geometric configurations strongly influence BA plaque distribution. In the present study, BA plaques were evenly distributed in the Tuning Fork geometry, which was consistent with the previous study (7). In the Tuning Fork geometry, the BA flow is roughly parallel and the velocity profile peak is rather central in the basilar artery, and the hemodynamic distribution is simple. However, in the vertebrobasilar arteries with Walking, Lambda, and No Confluence configurations, BA plaques were more frequently occurred at the lateral wall, from where the penetrating artery arose (all *P* ≤ 0.05). A study by Kim et al. (13) demonstrated that greater mid-BA angulation may enhance lateral plaque formation, and greater BA-VA angulation may enhance posterior plaque formation. In our research results, the AP-Mid-BA angle in Walking (36.13° ± 18.27°), Lambda (24.84° ± 13.02°), and No Confluence (24.17° ± 10.54°) geometries were greater than in the Tuning Fork geometry (14.95° ± 11.66°, *P* = 0.001), and the greater AP-Mid-BA in the Walking, Lambda, and No Confluence configurations may be induced the high percentage of BA plaque in the lateral wall among the three geometries. Compared with patients without pontine infarction, patients with pontine infarction were more likely to have larger VA-BA angle (38.72° ± 26.01° vs. 26.59° ± 17.33°, *P* = 0.035), a larger VA-BA angle may be enhance the formation of posterior wall plaque, furthermore inducing pontine infarction. In addition, there were no significant differences in the VA-BA and Lateral-Mid-BA angles among the four vertebrobasilar geometries, which may be the reason that BA plaque distribution in the posterior wall among the four geometries have no significant differences.

Our study has many clinical implications. First, a previous study (11) reported that about two-thirds of BA plaques are located at the lateral and dorsal walls, where the penetrating artery arises, which have a high risk of pontine infarction, this suggests that BA plaque distribution may help us to assess the likelihood of ischemic stroke in the posterior circulation and reduce the risk of complications during stenting. We found about 50% of the total BA plaque located at the posterior wall where the penetrating artery arose in patients with pontine infarction, this is consistent with the previous study (11), which suggested that BA plaque in the posterior wall has a high risk of developing pontine infarction. Besides, we found that the incidence of BA plaque in the lateral wall was higher than in the anterior and posterior walls in patients without pontine infarction, and suggested that BA plaques in the upper lateral wall are unlikely to induce occlusion of the penetrating artery. Second, we demonstrated that geometric configurations and BA curvature angulation may influence the BA plaque distribution.

There are several limitations in this study. First, patients with vertebral artery atherosclerosis were not excluded from the study, VA is the upstream vessel of BA, thus, the hemodynamic effect of VA stenosis to BA atherosclerosis formation may be underestimated and simplified. Second, the analysis of the VBA geometry and the angle measurement were done manually from 2D plane of a 3D-reconstructed TOF-MRA. Third, the hemodynamic distribution in the four geometries was not evaluated. Further studies measuring the hemodynamic distribution to elucidate the mechanism by which the geometry influences plaque distribution is necessary. Fourth, we cannot entirely exclude the possibility that the pontine infarctions were caused by the branches or penetrating arteries lesions of BA in our patients. Finally, the sample size was small, only 13 patients have pontine infarction and only a single center was included, future studies recruiting larger populations are warranted. This study is an observational cross-sectional study which may be influenced by uncontrolled confounders.

## Conclusion

In conclusion, BA plaque is associated with the posterior circulation cerebral infarction (BA-PCA region). Furthermore, BA plaque in the posterior wall may be related to pontine infarction, and there are relationship between vertebrobasilar geometry and BA plaque distribution. Among the Walking, Lambda, and No Confluence geometries, BA plaques were often located at the lateral wall, while in the Tuning Fork geometry, the BA plaques were evenly distributed.

## Data availability statement

The raw data supporting the conclusions of this article will be made available by the authors, without undue reservation.

## Ethics statement

The studies involving human participants were reviewed and approved by the Ethics Committee of Union Medical College Hospital Affiliated to Fujian Medical University. The patients/participants provided their written informed consent to participate in this study.

## Author contributions

JZ analyzed the data and wrote the original draft. JZ, BS, RL, YT, and EZ collected the data. XZ analyzed the data and edited the manuscript. YX supervised the whole study and edited the manuscript. All authors read and approved the final manuscript.

## References

[1] Nouh A Remke J Ruland S. Ischemic posterior circulation stroke: a review of anatomy, clinical presentations, diagnosis, and current management. Front Neurol. (2014) 5:30. 10.3389/fneur.2014.00030 24778625 PMC3985033

[2] Amin-Hanjani S Pandey DK Rose-Finnell L Du X Richardson D Thulborn K. Effect of hemodynamics on stroke risk in symptomatic atherosclerotic vertebrobasilar occlusive disease. JAMA Neurol. (2016) 73:178–85. 10.1001/jamaneurol.2015.3772 26720181 PMC5274634

[3] Klein IF Lavallée PC Mazighi M Schouman-Claeys E Labreuche J Amarenco P. Basilar artery atherosclerotic plaques in paramedian and lacunar pontine infarctions: a high-resolution MRI study. Stroke. (2010) 41:1405–9. 10.1161/STROKEAHA.110.583534 20538696

[4] Bodle JD Feldmann E Swartz RH Rumboldt Z Brown T Turan TN. High-resolution magnetic resonance imaging: an emerging tool for evaluating intracranial arterial disease. Stroke. (2013) 44:287–92. 10.1161/STROKEAHA.112.664680 23204050 PMC3556720

[5] Watanabe H Yoshida K Akasaka T Hozumi T Yoshikawa J. Intravascular ultrasound assessment of plaque distribution in the ostium of the left anterior descending coronary artery. Am J Cardio. (1996) 78:827–9. 10.1016/S0002-9149(96)00431-6 8857492

[6] Xu WH Li ML Gao S Ni J Zhou LX Yao M. Plaque distribution of stenotic middle cerebral artery and its clinical relevance. Stroke. (2011) 42:2957–9. 10.1161/STROKEAHA.111.618132 21799160

[7] Yu J Zhang S Li ML Ma Y Dong YR Lou M. Relationship between the geometry patterns of vertebrobasilar artery and atherosclerosis. BMC Neurol. (2018) 18:83. 10.1186/s12883-018-1084-6 29895279 PMC5996488

[8] Wake-Buck Amanda K Gatenby JC Gore JC. Hemodynamic characteristics of the vertebrobasilar system analyzed using MRI-based models. PLoS ONE. (2012) 7:e51346. 10.1371/journal.pone.0051346 23251503 PMC3519605

[9] Zheng JM Sun B Lin RL Teng YQ Zhao XH Xue YJ. Association between the vertebrobasilar artery geometry and basilar artery plaques determined by high-resolution magnetic resonance imaging. BMC Neurosci. (2021) 22:20. 10.1186/s12868-021-00624-5 33765922 PMC7992992

[10] Mendelson Scott J Prabhakaran S. Diagnosis and management of transient ischemic attack and acute ischemic stroke: a review. JAMA. (2021) 325:1088–98. 10.1001/jama.2020.26867 33724327

[11] Yu J Li ML Xu YY Wu SW Lou M Mu XT. Plaque distribution of low-grade basilar artery atherosclerosis and its clinical relevance. BMC Neurol. (2017) 17:8. 10.1186/s12883-016-0785-y 28068949 PMC5223551

[12] Hong JM Chung CS Bang OY Yong SW Joo IS Huh K. Vertebral artery dominance contributes to basilar artery curvature and peri-vertebrobasilar junctional infarcts. J Neurol Neurosurg Psychiatry. (2009) 80:1087–92. 10.1136/jnnp.2008.169805 19414436 PMC2735647

[13] Kim BJ Lee KM Kim HY Kim YS Koh SH Heo SH. Basilar artery plaque and pontine infarction location and vascular geometry. J Stroke. (2018) 20:92–8. 10.5853/jos.2017.00829 29402062 PMC5836573

