# Supplementary material for: Basilar artery plaque distribution is associated with pontine infarction and vertebrobasilar artery geometry
Source: Front Neurol. 2023 Mar 13;14:1079905. doi: 10.3389/fneur.2023.1079905 (PMC10040971; doi:10.3389/fneur.2023.1079905)
Supplement: Supplementary file 1 [file Data_Sheet_1.pdf]

Basilar artery plaque distribution is associated with pontine infarction and vertebrobasilar artery geometry

Supplementary figure 1:

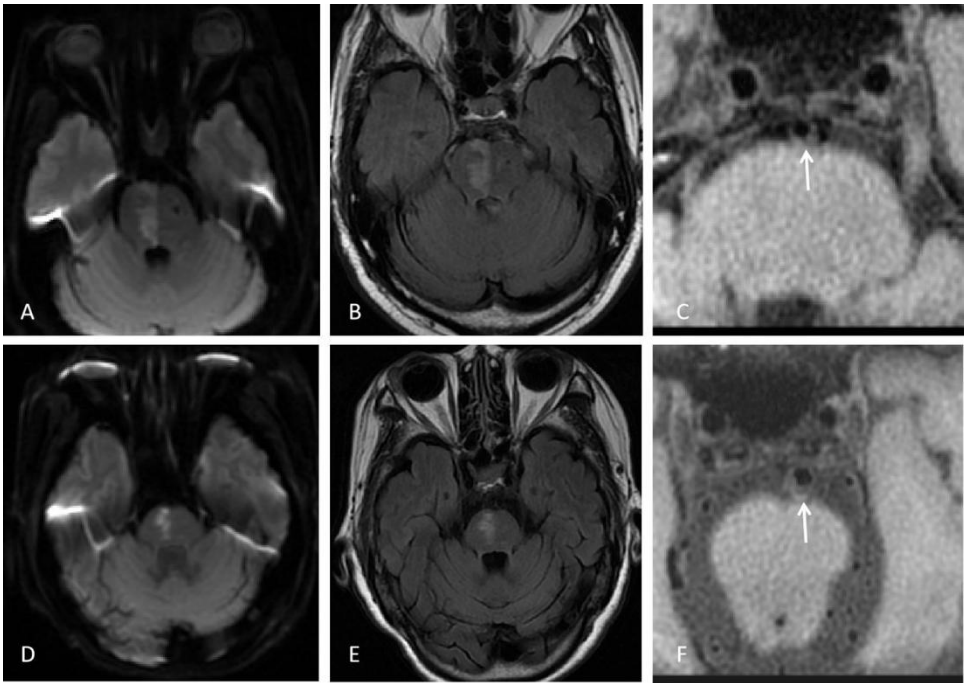

Patient one (A-C) , man, 67y, a paramedian pontine infarction was show on DWI (A) and T2WI-FLAIR (B), a posterior wall BA plaque was seen on the corresponding plane (C). Patient two (D-F), female, 75y, a paramedian pontine infarction was show on DWI (D) and T2WI-FLAIR (E), a posterior wall BA plaque was seen on the corresponding plane (F).
